# Improvement of Terahertz Wave Radiation for InAs Nanowires by Simple Dipping into Tap Water

**DOI:** 10.1038/srep36094

**Published:** 2016-10-26

**Authors:** Dong Woo Park, Young Bin Ji, Jehwan Hwang, Cheul-Ro Lee, Sang Jun Lee, Jun Oh Kim, Sam Kyu Noh, Seung Jae Oh, Sang-Hoon Kim, Tae-In Jeon, Kwang-Un Jeong, Jin Soo Kim

**Affiliations:** 1 Division of Advanced Materials Engineering & Research Center of Advanced Materials Development, Chonbuk National University, Jeonju 54896, Republic of Korea; 2 Materials Genome Center, Korea Research Institute of Standards and Science, Daejeon 34113, Republic of Korea; 3 Medical Convergence Research Institute, College of Medicine, Yonsei University, Seoul 03722, Republic of Korea; 4 Applied Electromagnetic Wave Research Center, Korea Electrotechnology Research Institute, Ansan 15588, Republic of Korea; 5 Division of Electrical and Electronics Engineering, Korea Maritime and Ocean University, Busan 49112, Republic of Korea; 6 Department of Polymer-Nano Science and Technology, and Polymer Materials Fusion Research Centre, Chonbuk National University, Jeonju 54896, Republic of Korea

## Abstract

We report improvement of terahertz (THz) wave radiation for Si-based catalyst-free InAs nanowires (NWs) by simple dipping into tap water (DTW). In addition, the possibility of using InAs NWs as a cost-effective method for biomedical applications is discussed by comparison to bulk InAs. The peak-to-peak current signals (PPCSs) of InAs NWs measured from THz time-domain spectroscopy increased with increasing NW height. For example, the PPCS of 10 μm-long InAs NWs was 2.86 times stronger than that of 2.1 μm-long NWs. The THz spectra of the InAs NWs obtained by applying a fast Fourier transformation to the current signals showed a main frequency of 0.5 THz, which can be applied to a variety of medical imaging systems. After the DTW process, structural variation was not observed for 2.1 μm-long InAs NWs. However, the top region of several InAs NWs with heights of 4.6 and 5.8 μm merged into a conical structure. InAs NWs with a height of 10 μm resulted in a bundle feature forming above the conical shape, where the length of bundle region was 4 μm. After the DTW process, the PPCS for 10 μm-long InAs NWs increased by 15 percent compared to that of the as-grown case.

The terahertz (THz) gap positioned between the far-infrared and microwave regions in the electromagnetic spectra has remained one of the most challenging regions to analyze in terms of fundamental physics as well as device applications^1^,^2^,^3^,^4^. This THz frequency range is of particular interest for a variety of medical imaging applications, including skin and breast cancer margin detection, burn wound imaging, skin hydration monitoring, and corneal hydration measurement^5^. To obtain the effective biomedical information regarding THz technology, suitable THz sources and detectors are required. Recently, III-V semiconductors, such as low-temperature grown (LT) GaAs, LT-InGaAs, and LT-InGaAs/InAlAs multiple quantum wells, have been actively studied for THz applications by using a photoconductive mechanism under an external direct-current (DC) bias^6^,^7^,^8^,^9^,^10^.

Over the past few years, a nanowire (NW)-based THz source is expected to be adequately applied to molecular fingerprint applications due to the larger effective interface between NWs and biological features compared to that of epilayers. G. B. Jung *et al*. demonstrated the height dependence of Si NWs formed on Si by a metal-assisted etching process, where the THz generation increased with increasing height from 0.3 to 3 μm^11^. J. J. Ibanes *et al*. reported THz wave radiation from Au-catalyst GaAs-AlGaAs core-shell structures, where the radiation was enhanced by applying a magnetic field^12^. K. Peng *et al*. demonstrated a photoconductive THz detector using single GaAs-AlGaAs core-shell NWs^13^. In addition, InAs and InSb have been used for THz emission by utilizing the photo-Dember (p-D) effect^14^,^15^,^16^,^17^. For Ga(In)As epilayers, DC bias conditions for THz wave radiation are required for carrier acceleration. However, a DC bias is not usually necessary for InAs because of the large difference of the mobility between electrons and holes generated from an external femto-second (fs) excitation source^14^,^17^. In a previous report on p- and n-type InAs wafers by K. Liu *et al*.^17^, THz wave radiation depending on the carrier concentration indicated that a low carrier concentration is more suitable for THz emission. This is related to additional doped carriers significantly weakening or “screening” the p-D field, consequently reducing the THz wave radiation. Usually, the THz wave radiation from InAs bulk is in the range from 0 to 7 THz^14^. However, the amplitude curve with THz frequency obtained from the fast Fourier transformation (FFT) of the current signals of the time-domain waveform is not uniform. For specific applications such as biomedical analysis and imaging systems, more reliable and reproducible characteristic curves of the THz wave radiation in the frequency range around ~0.5 THz are needed. According to previous reports, THz devices with InAs NWs showed a narrower emission range in frequency response compared to that of bulk InAs. However, the THz spectrum of InAs NWs is sufficiently adequate for biomedical analysis, such as *in vivo* and *ex vivo* diagnoses around ~0.5 THz, with a relatively large effective contact area compared to the bulk^18^,^19^,^20^. Compared to commercial InAs wafers, the contact area of NWs with biomedical specimens can be enhanced due to the additional side-wall contact, resulting in an increase of the reliability level of information. In addition, from the economical point of view for commercial applications, InAs NWs on Si have significant advantages compared to InAs bulk. That is, if THz devices use Si-based InAs NW, single-use indicators can be provided for biomedical specimens. Recently, D. V. Seletskiy *et al*. demonstrated THz wave radiation around 2 THz using InAs NWs formed on Au catalysts. They reported that the amplitude of the THz spectra for InAs NWs with a fill factor of 0.03, defined as the ratio of the total area of the top surfaces for InAs NWs to the flat surface, decreased by only about 50% compared to that of a slightly n-doped InAs wafer^21^. A. Arlauskas *et al*. analyzed THz wave radiation using InAs NWs formed on hole-array patterned SiO_2_/Si templates, where the amplitude was 60% of that of p-type bulk InAs (~10^18^ cm^−3^)^22^. In most previous works, InAs NWs on Si substrates were formed by using metallic catalysts such as Au and Ni^23^,^24^,^25^. Metallic catalysts may create deep levels in the energy bandgap mainly due to chemical contamination, resulting in potential degradation of the electrical and optical properties of InAs NWs. Therefore, it is better to remove catalysts or chemical patterns used for the nucleation sites of NWs in order to increase the THz wave radiation of InAs NWs. In addition, for commercial applications such as molecular fingerprints and imaging, a stronger THz wave radiation of InAs NWs formed on Si is required.

In this paper, we report the height-dependent THz wave radiation characteristics of catalyst-free InAs NWs formed on Si(111). In addition, there was enhancement of the peak-to-peak current signal (PPCS) of the THz time-domain spectroscopy (TDs) by simply dipping InAs NWs into normal tap water (DTW). After the DTW process, several catalyst-free InAs NWs were merged into a conical feature via van der Waals bonding. After forming the conical structures, they coalesced again to form larger bundle features for relatively long InAs NWs. The formation of the bundle structures of the InAs NWs led to a reduction of the effective trapping states of the carriers, resulting in improvement of the THz radiation. Here, we suggest a possible way to improve the THz characteristics of NW-based structures by a simple DTW process.

## Results

**Figure 1:**
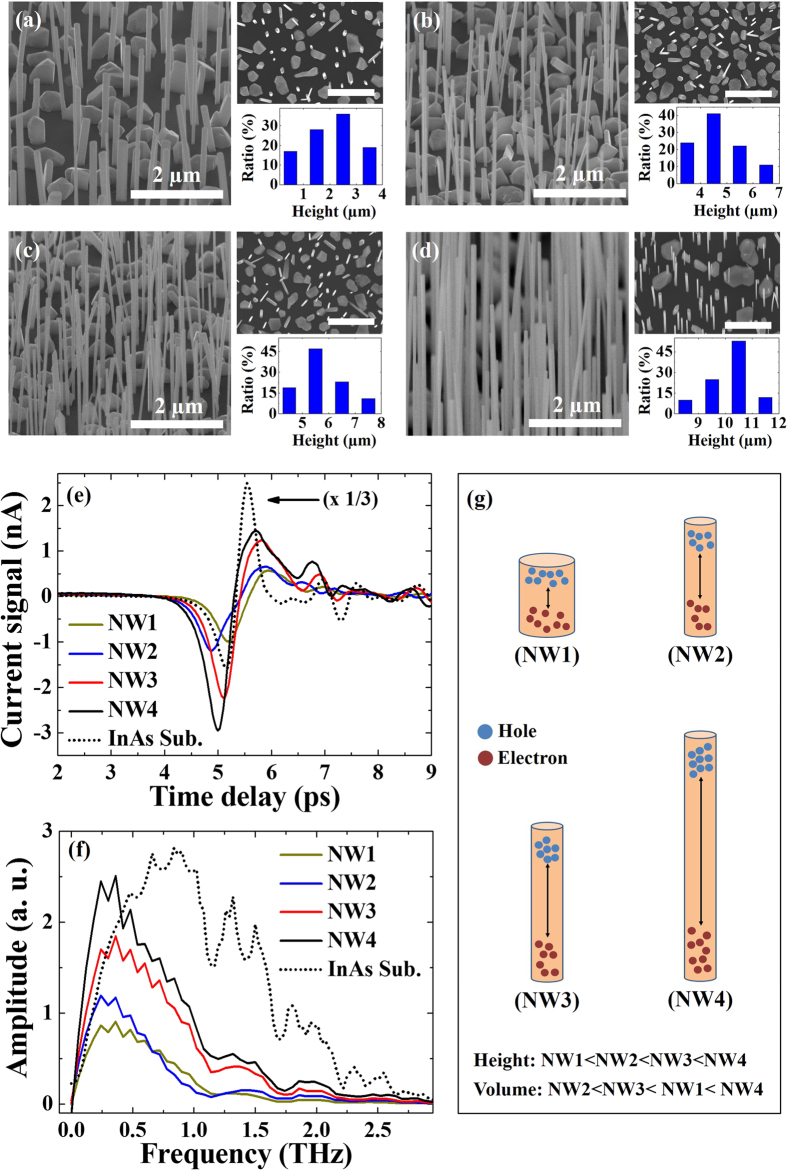
FE-SEM images of as-grown InAs NWs in the (**a**) NW1, (**b**) NW2, (**c**) NW3, and (**d**) NW4 samples, where each right-hand side shows a plan-view image (upper image, scale bar: 2 μm) and height distribution (bottom graph) of InAs NWs, (**f**) THz spectra obtained by applying FFT to the current signals of InAs NWs, and (**g**) schematic illustration of the carrier dynamics.

The InAs NW samples were grown on Si(111) substrates at different V/III ratios defined as the flux ratio of the group-V element (As) to the group-III element (In). As described in Table 1, V/III ratios for the formation of the InAs NWs were 200 (NW1 sample), 300 (NW2 sample), and 400 (NW3 and NW4 samples). Figures 1(a–d) show tilt-view FE-SEM images of the NW1, NW2, NW3, and NW4 samples, respectively. The structural parameters of the InAs NWs were summarized in Table 2. With increasing V/III ratio during the formation of InAs NWs, the height of the NWs increased. This is attributed to the fact that large amount of As adatoms at a relatively high V/III ratio can crystallize more In to generate InAs structures, leading to the growth of longer NWs. The upper right-hand side of each FE-SEM image shows a plan-view image, which was used to investigate the spatial density of the InAs NWs. As described in Table 2, the spatial densities of the InAs NW samples were almost the same. The bottom right-hand-side graph of each FE-SEM image shows the height distribution of InAs NWs. The standard deviations of the height for the NW samples were also summarized in Table 2. The NW4 sample shows the smallest value in the standard deviations of the samples. This indicates that the InAs NWs for the NW4 sample are relatively uniform compared to the other NW samples. More details of the structural characteristics of InAs NWs depending on the growth parameters are shown in our previous report^26^. Figure 1(e) shows the THz current signals of the InAs NWs with respect to the time delay for the NW samples. As a reference, the spectrum for a commercial undoped InAs wafer (Wafer Tech) was measured, where the PPCS was calculated to be 13.08 nA. The PPCSs for the NW1, NW2, NW3, and NW4 samples were measured to be 1.54, 1.84, 3.44, and 4.40 nA, respectively. To compare with the structural properties, PPCSs of the InAs NW samples were described in Table 2. The PPCS for the NW4 sample was measured to be 33% of the InAs substrate. However, if we consider the fill factor of 0.005 for InAs NWs, the amplitude of the PPCS is meaningful. In a previous report by D. V. Seletskiy *et al*., the reduction rate for the PPCS for the InAs NWs formed on Au-catalysts with a fill factor of 0.03 was 50% compared to that of an intentionally low n-doped InAs wafer^21^. Even though the fill factor of InAs NWs in this work is 1/6 that of the previous report, the reduction rate is relatively small. In addition, the PPCS usually becomes low with increasing carrier concentration of InAs wafers^17^. Here, we used an undoped InAs wafer for comparison and as a result, the reference value was expected to be higher than that of the previous work. Also, while they used 20 μm-long InAs NWs^21^, the longest NWs in our work were only 10 μm. Based on these considerations, the catalyst-free InAs NWs surely give more effective media for THz wave radiation. This improvement of the THz radiation characteristics of catalyst-free InAs NWs is attributed to a reduction of carrier trapping sites related to metal catalysts. Figure 1(f) shows the THz spectra of InAs NWs with respect to their height obtained by applying FFT to the TDs current signals. An undoped InAs substrate was also considered for comparison. The amplitude of the THz spectra of the NW samples increased with increasing height of the InAs NWs at a frequency of ~0.5 THz. In particular, the amplitude of the THz spectrum for the NW4 sample is higher than that of the InAs substrate in the low frequency region (~0.5 THz). Even though the bandwidth for InAs NWs is smaller than that of an InAs substrate, InAs NWs can be applied to specific applications such as molecular fingerprinting and imaging systems operating at a frequency of ~0.5 THz. For example, biomedical specimens such as basal cell carcinoma and normal human skin tissue are very sensitive to the frequency range of around 0.5 THz^5^,^27^,^28^. Also, the effective contact area with a certain biomedical specimen for the NW4 sample was calculated to be 2.67 times higher than that of the InAs substrate. From this point of view, InAs NWs on Si have significant advantages compared to bulk InAs for commercial applications. Moreover, since Si-related technology is well-developed and it is relatively cheap to prepare Si-based InAs NWs compared to InAs wafers, single-use indicators for biomedical specimens using Si-based InAs NWs are possible. Figure 1(g) shows a schematic illustration of the carrier behaviors of InAs NWs with different heights. In Table 2, the volume of the NW1 sample was larger than those of the NW2 and NW3 samples because of the relatively large width. As shown in Fig. 1(b–d), the average widths of the InAs NWs in the NW2, NW3, and NW4 samples were similar. As a result, the volume of the NW4 sample was relatively large among the three samples because of the NW height. Even though the effective volume of the NW1 sample is larger than those of the other samples, the PPCS and THz spectral amplitude of the InAs NWs were relatively low, which can be attributed to the low p-D effect. That is, the spatial separation between electrons and holes could be increased with increasing height of the NWs, resulting in an increase of the dipole strength within a certain range. In addition, the PPCS and THz spectral amplitude of the NW4 sample were relatively large compared to those of the NW2 and NW3 samples. This indicates that the dependence on the NW height instead of volume is more effective for THz wave radiation of InAs NWs.

**Figure 2:**
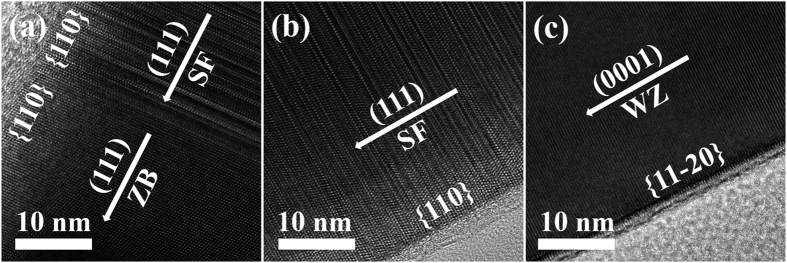
HR-TEM images of the (**a**) region exhibiting ZB and SF (bottom of an InAs NW), (**b**) region exhibiting SF only (center), and (**c**) region exhibiting WZ only (top).

Figure 2 shows cross-sectional Cs-TEM images of the InAs NWs. For the catalyst-free InAs NWs, nucleation randomly appeared on various sites of the substrate because catalysts and patterns were not used. Figure 2(a) is a high-resolution TEM (HR-TEM) image of the region exhibiting both zincblende (ZB) and stacking faults (SF), in which the growth direction and side facet were (111) and {110}, respectively. Figure 2(b) is a TEM image of the region with SF positioned between ZB and wurtzite (WZ). The WZ structures shown in Fig. 2(c) were mostly observed at the upper region of the InAs NW, where the growth direction and side facet were consequently changed to (0001) and {11–20}. The change of the crystal structure of the InAs NW from ZB to WZ via SF can be explained by the fact that the WZ structure is more stable for InAs NWs due to the relatively low surface energy.

**Figure 3:**
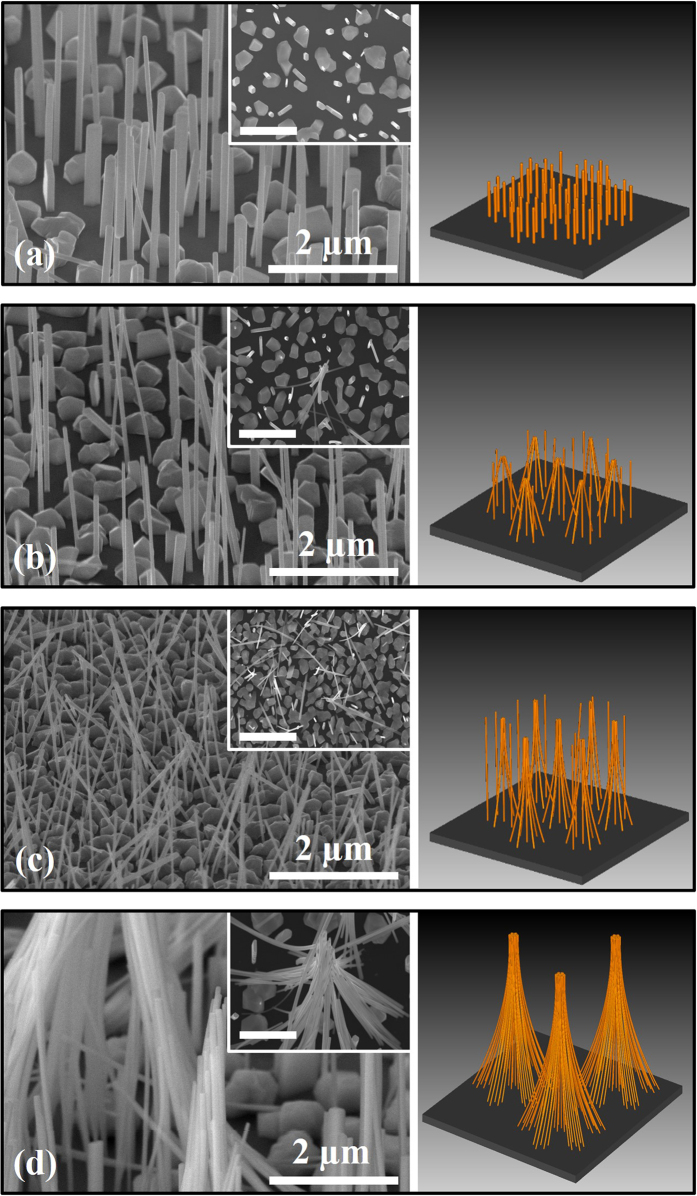
FE-SEM images of InAs NWs in the (**a**) NW1, (**b**) NW2, (**c**) NW3, and (**d**) NW4 samples after the DTW process. The inset in each FE-SEM image shows an expanded image, where the scale bar is 2 μm. The right-hand side is a schematic of the formation of InAs conical and bundle structures.

Figures 3(a–d) show three-dimensional FE-SEM images of the InAs NWs in the NW1, NW2, NW3, and NW4 samples, respectively, subjected to the DTW process. For the NW1 sample with a height of 2.1 μm, there was no merging effect among the NWs after the DTW process. This can be explained by the fact that the length of the InAs NWs was not enough to bend and to make a coalescent structure. However, the top region of several InAs NWs in the NW2 and NW3 samples coalesced into a conical structure. It seems like that some of the InAs NWs were randomly collapsed. However, the coalescent phenomenon of the InAs NWs was obviously caused by the simple DTW process, if we consider the images for as-grown InAs NWs shown in Fig. 1(b,c). For relatively long NWs, the bundling of InAs NWs was more clearly observed in Fig. 3(d). For the NW4 sample with a height of 10 μm, several conical structures again merged into a much bigger bundle feature, where the length of the bundle region above the conical point was 4 μm. The inset in each FE-SEM image shows an expanded image used to investigate the structure of the coalescent NWs, where the scale bar is 2 μm. Recently, the bundling or self-attraction has been reported for Si, ZnO, and GaAs NWs^29^,^30^,^31^,^32^ during growth or by post-growth treatments. However, there is no report on the bundling of InAs NWs. To the best of our knowledge, this is the first observation on the bundle structures of InAs NWs. In addition, it is noteworthy that this phenomenon was repeatedly obtained and was observed even for InAs NWs grown by a metal-organic chemical-vapor deposition (MOCVD) [the images at “Supplementary information” show the bundling of MOCVD-grown InAs NWs by the DTW process]. The merging phenomena among the adjacent NWs in the NW2, NW3, and NW4 samples can be explained by van der Waals bonding^32^,^33^,^34^. A conceptual structure estimated from the FE-SEM results is schematically shown in the right-hand side of each FE-SEM image. The NW densities contributing to conical structures were measured to be 4 × 10^7^, 6.5 × 10^7^, and 9.8 × 10^7^ cm^−2^ for the NW2, NW3, and NW4 samples, respectively. That is, the number of InAs NWs contributing to the conical structures increased with increasing height of the NWs. This is related to the increase of the bending radius with increasing height of the NWs. The bending radius of the NW4 sample was calculated to be 6 μm, which is higher than those of the NW2 (0.42 μm) and NW3 (2.4 μm) samples. When adjacent InAs NWs adhere at a certain critical height, the system is relatively stable because the adhesion among their contacting surfaces is equal (at least) to the elastic force of their deformation or bending.

**Figure 4:**
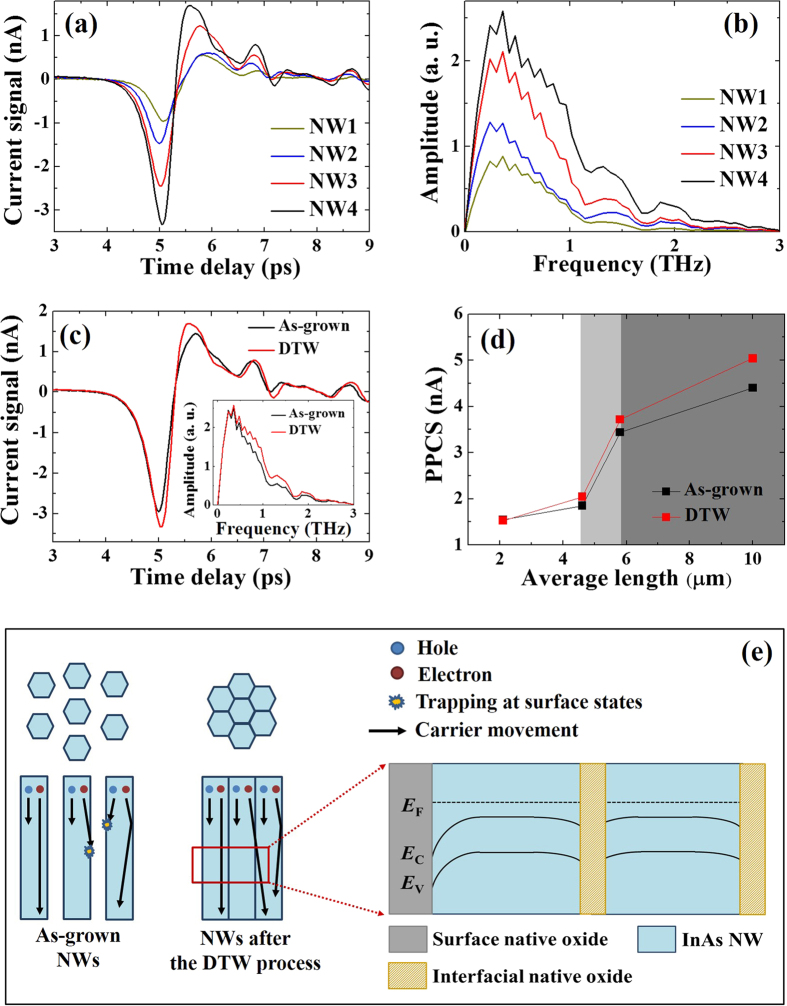
(**a**) TDs current signals, (**b**) THz spectra of InAs NW samples, and (**c**) comparison of the current signals of the NW4 sample before and after the DTW process. (**d**) Summary of the PPCS of the NW samples before and after the DTW process, and (**e**) schematic illustration of the improvement of THz wave radiation for the bundle structure of the InAs NWs. The band structure of InAs NWs consisting of bundle structure was schematically shown, where the bending of *E*_C_ and *E*_V_ bands surely occurs.

Figure 4(a,b) show the THz current signals of the InAs NW samples after the DTW process, where the PPCSs of the NW1, NW2, NW3, and NW4 samples were measured to be 1.53, 2.04, 3.71, and 5.04 nA, respectively. By applying FFT to the current signals, the THz spectra were obtained, as shown in Fig. 4(b), where the center and cut-off frequencies for the NW samples were 0.5 and 1.5 THz, respectively. The amplitude of the THz spectra increased with increasing height of the InAs NWs, which is similar to that of the as-grown samples. Figure 4(c) shows the THz current signal for the NW4 sample before and after the DTW process for comparison, where the inset shows the corresponding THz spectra. Figure 4(d) shows the comparative summary of the PPCSs of the NW samples with the heights of the InAs NWs before and after the DTW process. The rate of the PPCS of InAs NWs subjected to the DTW process increased with increasing height of the InAs NWs. The amplitude of the PPCS for the NW4 sample after the DTW process was 1.15 times higher than that of the as-grown case (4.40 nA). This can be explained by the reduction of carrier trapping at the surface states and an increase in tunneling probability of carriers among neighboring NWs^35^,^36^,^37^. Native oxides, such as InOx and AsOx, are surely formed at the surface of InAs NWs, which can be worked as recombination centers of carriers. As shown in the left-hand side of Fig. 4(e), the carriers generated by supplying an fs-laser can move toward the bottom region of the InAs NWs. During the carrier movement, there is a possibility for some of the carriers to be captured at the surface states caused by the existence of native oxides, resulting in degradation of the current signals of InAs NWs before the DTW process. However, the possibility for the carriers to be trapped at the surface states is reduced because of the formation of bundle structures of InAs NWs after the DTW process. For a more detailed discussion of carrier movement between neighboring InAs NWs, energy band structure with Fermi level (*E*_F_) for a bundle structure of InAs NWs was schematically shown in the right-hand side of Fig. 4(e). The bending of the conduction (*E*_C_) and valence (*E*_V_) bands usually occurs due to termination of carrier wave-functions at the interface to the native oxide. R. Timm *et al*. investigated the structure and chemical composition (InOx, AsOx, As-As metallic bonds, etc.) of a native-oxide layer between InAs NWs and high-ĸ films^38^. Upon deposition of high-ĸ films, reduction of interfacial native oxide was observed. Similarly, the native oxide between adjacent InAs NWs in this work may be reduced, conceptually illustrated in Fig. 4(e), where the interfacial native oxide is relatively thinner than the surface native oxide. As a result, the recombination probability of carriers at the native oxide could be reduced. If we consider the reduction of the interfacial oxide layer and a back-to-back configuration of two NW-interfacial native oxide junctions, the band bending is expected to be alleviated between adjacent NWs, resulting in an increase in tunneling probability of carriers inside NW bundles. Also, carrier movement between InAs NWs can occur via As-As metallic bonds consisting of native oxide. By these phenomena, an improvement in the THz emission of InAs NW bundles was observed after the simple DTW process. To determine dominant or significant parameter(s) on the improvement in THz characteristics of InAs NW bundles, theoretical calculation will be performed, which will be discussed elsewhere.

## Discussion

The THz radiation characteristics of catalyst-free InAs NWs formed on Si(111) were investigated by changing the structural dimensions and by adopting a simple DTW process. The PPCS of the InAs NWs obtained from THz TDs increased from 1.54 to 4.40 nA as the NW height increased from 2.1 to 10 μm. The increase of the PPCS of the InAs NWs depending on the height of the NWs was related to the enhancement of the dipole generation via the increase in the height of the NWs. For the 2.1 μm long InAs NWs, there was no merging effect between adjacent NWs after the DTW process. However, the top region of several InAs NWs with heights of 4.6 and 5.8 μm merged into conical bundle structures via van der Waals forces. For the InAs NWs with a height of 10 μm, the conical bundle structures again merged into larger bundle features, where the length contributing to the bundle region was 4 μm. After the DTW process, the PPCS of the InAs NWs with a height of 10 μm increased to 5.04 nA, which was 1.15 times higher than that of the as-grown InAs NWs. The improvement of the THz characteristics of InAs NWs by the DTW process can be explained by the reduction of carrier trapping at the surface states and the increase in tunneling probability of carriers between adjacent NWs due to the formation of bundle features.

In terms of applications for liquid-based specimen such as human blood, devices based on nano-structures including NWs should be sometimes dipped into liquid phase. After dipping the device into a liquid, there is possibility for the nano-structures to be modified^32^,^39^, resulting in change in device characteristics. For example, the bundling of TiO_2_ nanotubes (NTs) arose during evaporative drying of wetted NT films from cleaning step^40^. The solar conversion efficiency of a dye-sensitized solar cell incorporating TiO_2_-NT bundles was degraded compared to that containing bundle-free NTs. Considering the promising biomedical applications of InAs NWs and previous results, the improvement in the THz characteristics of InAs NW bundles obtained by the simple DTW process is meaningful.

## Methods

The InAs NWs were grown on Si(111) substrates using Riber32P molecular-beam epitaxy (MBE) with solid sources. Before the formation of the InAs NWs, Si(111) substrates were chemically cleaned using a standard wet etching process with acetone, methanol, isopropyl alcohol, and deionized water (DI water). A deoxidation process was then performed using a hydrofluoric acid (HF) solution [HF (1):DI water (20)]. After the chemical deoxidation process, the Si(111) substrates were immediately loaded into an MBE load-lock chamber. The growth rate of the InAs NWs was 0.01 Å/second, which was estimated from nominal two-dimensional (2D) epitaxial growth. The growth temperature for the InAs NWs was fixed at 430 °C. The V/III ratios for the formation of the InAs NWs were 200 (NW1 sample), 300 (NW2 sample), and 400 (NW3 and NW4 samples), which were obtained by controlling the As flux at a fixed In beam equivalent pressure of 3 × 10^−8^ torr. The growth times of the NW1, NW2, and NW3 samples were 60 minutes. The growth parameters for the NW4 sample were exactly the same as those for the NW3 sample, except for the growth time (120 minutes). The growth details of the NW samples are shown in Table 1. In the DTW process, the InAs NWs were dipped into normal tap water for 1 minute. After the DTW process, the InAs NWs were dried in air after blowing the residual water using nitrogen gas. The external dimensions of the InAs NWs were measured by using a field-emission scanning electron microscope (FE-SEM, Hitachi S-4800). The internal structural properties of the InAs NWs were measured using a Cs-corrected field emission transmission electron microscope (Cs-TEM, Jem-arm200f). THz wave radiation for the samples was measured by using THz TDs. A mode-locked Ti:sapphire laser (Spectra-physics) with a pulse duration of 80 fs at a central wavelength of 800 nm was used to obtain the THz wave radiation. A circular laser beam with a diameter of 1.0 mm was incident on the sample surfaces at an angle of 45°. The number of InAs NWs in the laser spot was estimated to be 1.3 × 10^6^. The THz signals were measured using an LT-GaAs receiver with the dipole antenna under a laser power of 11 mW. In the initial stage of THz measurements on the InAs NW samples, we confirmed the uniformity in the THz characteristics of InAs NWs over an entire 2-inch wafer. The THz signals were randomly measured at five different points on a 2-inch wafer. The shape and intensity of the THz signals were almost same. [The THz signals of 10 μm-long InAs NWs measured at five different points on a 2-inch wafer are shown at “Supplementary information”]. The TDs current signals of the InAs NWs were converted into frequency spectra by applying the FFT.

## Additional Information

**How to cite this article**: Park, D. W. *et al*. Improvement of Terahertz Wave Radiation for InAs Nanowires by Simple Dipping into Tap Water. *Sci. Rep.* **6**, 36094; doi: 10.1038/srep36094 (2016).

**Publisher’s note:** Springer Nature remains neutral with regard to jurisdictional claims in published maps and institutional affiliations.

## Supplementary Material

Supplementary Information

## Figures and Tables

**Table 1:** Growth parameters of catalyst-free InAs NWs formed on Si(111).

Sample	In flux (torr)	As flux (torr)	V/III ratio (Arb. units)	Growth time (minutes)
NW1	3 × 10^−8^	6.0 × 10^−6^	200	60
NW2		9.0 × 10^−6^	300	60
NW3		1.2 × 10^−5^	400	60
NW4		1.2 × 10^−5^	400	120

**Table 2:** Summary on the structural properties and the PPCSs of InAs NW samples.

Sample	Average height (μm)	Average width (nm)	Spatial density (cm^−2^)	Standard deviation of height (μm)	Average volume (cm^3^)	PPCS (nA)
NW1	2.1	150	1.5 × 10^8^	0.98	3.7 × 10^−14^	1.54
NW2	4.6	85	1.2 × 10^8^	0.93	2.6 × 10^−14^	1.84
NW3	5.8	80	1.2 × 10^8^	0.89	2.9 × 10^−14^	3.44
NW4	10	80	1.2 × 10^8^	0.83	5.0 × 10^−14^	4.40
