# Supplementary material for: Improvement of Terahertz Wave Radiation for InAs Nanowires by Simple Dipping into Tap Water
Source: Sci Rep. 2016 Oct 26;6:36094. doi: 10.1038/srep36094 (PMC5080572; doi:10.1038/srep36094)
Supplement: Supplementary Information [file srep36094-s1.doc]

Supplementary information

Improvement of Terahertz Wave Radiation for InAs Nanowires by Simple Dipping into Tap Water

Dong Woo Park^1^, Young Bin Ji^3^, Jehwan Hwang^2^, Cheul-Ro Lee^1^, Sang Jun Lee^2^, Jun Oh Kim^2^, Sam Kyu Noh^2^, Seung Jae Oh^3^, Sang-Hoon Kim^4^, Tae-In Jeon^5^, Kwang-Un Jeong^6^, and Jin Soo Kim^1^

^1^Division of Advanced Materials Engineering & Research Center of Advanced Materials Development, Chonbuk National University, Jeonju 54896, Republic of Korea

^2^Materials Genome Center, Korea Research Institute of Standards and Science, Daejeon 34113, Republic of Korea

^3^Medical Convergence Research Institute, College of Medicine, Yonsei University, Seoul 03722, Republic of Korea

^4^Applied Electromagnetic Wave Research Center, Korea Electrotechnology Research Institute, Ansan 15588, Republic of Korea

^5^Division of Electrical and Electronics Engineering, Korea Maritime University, Busan 49112, Republic of Korea

^6^Department of Polymer-Nano Science and Technology, and Polymer Materials Fusion Research Centre, Chonbuk National University, Jeonju 54896, Republic of Korea

Correspondence and requests for materials should be addressed to Jin Soo Kim (email: kjinsoo@jbnu.ac.kr) or Sam Kyu Noh (email: sknoh@kriss.re.kr)

**S1. MOCVD Growth of catalyst-free InAs NWs**

Catalyst-free InAs NWs were grown on p-type Si(111) substrates by using metal-organic chemical vapor deposition (MOCVD, AIXTRON Inc.) with a horizontal reactor. Si(111) substrates were chemically cleaned using a standard wet-etching process with acetone, methanol, isopropyl alcohol, and deionized water (DI water). A deoxidation process was then performed using a hydrofluoric acid (HF) solution [HF (1):DI water (20)]. After the chemical deoxidation process, the Si(111) substrates were immediately loaded into an MOCVD reactor. The InAs NWs were grown at the substrate temperature of 570 °C. The growth time for InAs NWs was three hours. **Figure S1** shows the FE-SEM (Hitachi S-4800) images of as-grwon InAs NWs (by MOCVD) with different zoom magnifications and directions. The averge height and dimeter of InAs NWs were meausred to be 30 μm and 200 nm, respectively.


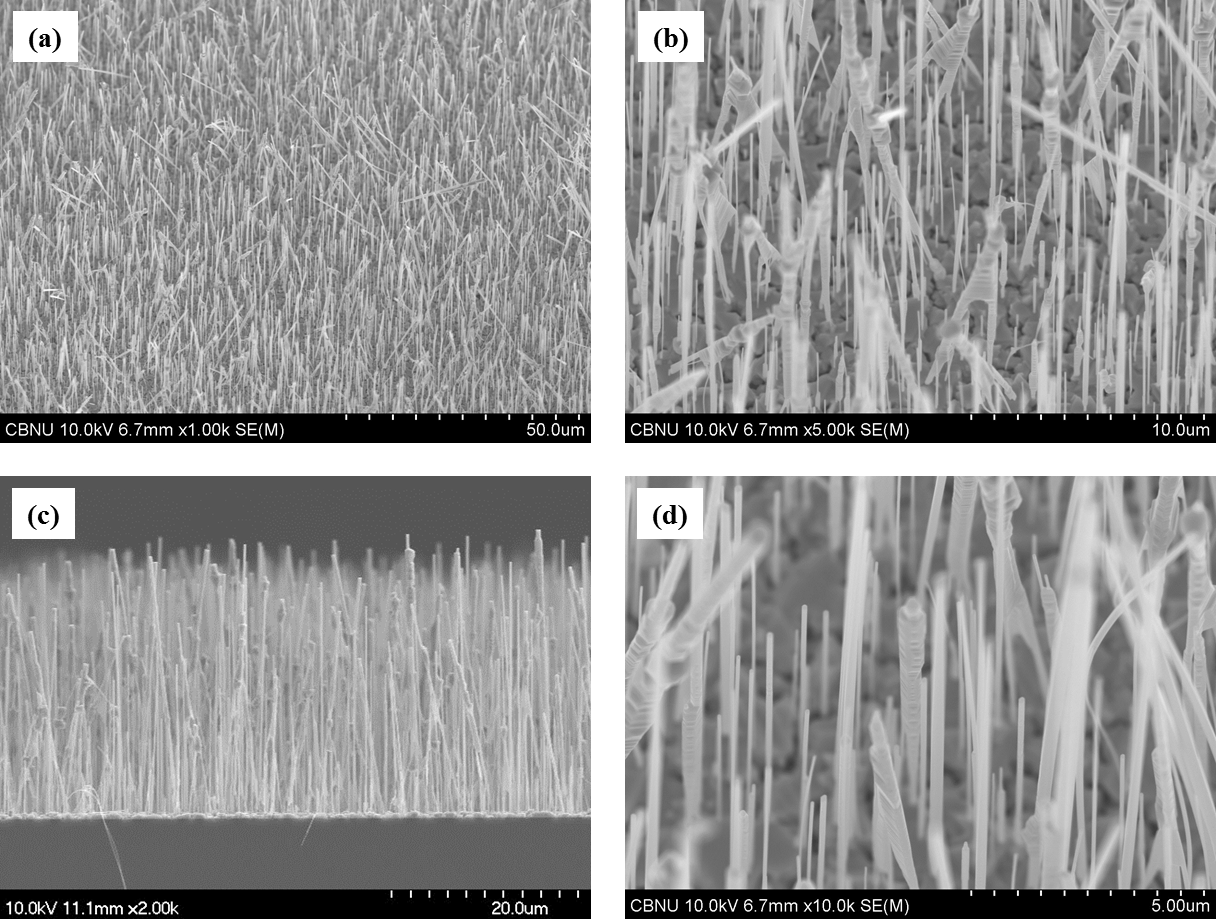


**Figure S1** | FE-SEM images of as-grown InAs NWs with different magnifications and directions.

**S2. MOCVD-grown InAs NWs subjected to the DTW process.**

For the DTW process, the MOCVD-grown InAs NWs were dipped into normal tap water for one minute. After the DTW process, the InAs NWs were dried in air after blowing the residual water using nitrogen gas. **Figure S2** shows the FE-SEM images of InAs NWs after the DTW process. The bundle structures of MOCVD-grown InAs NWs were clearly observed, which is similar to the MBE-grown InAs NWs subjected to the DTW process.


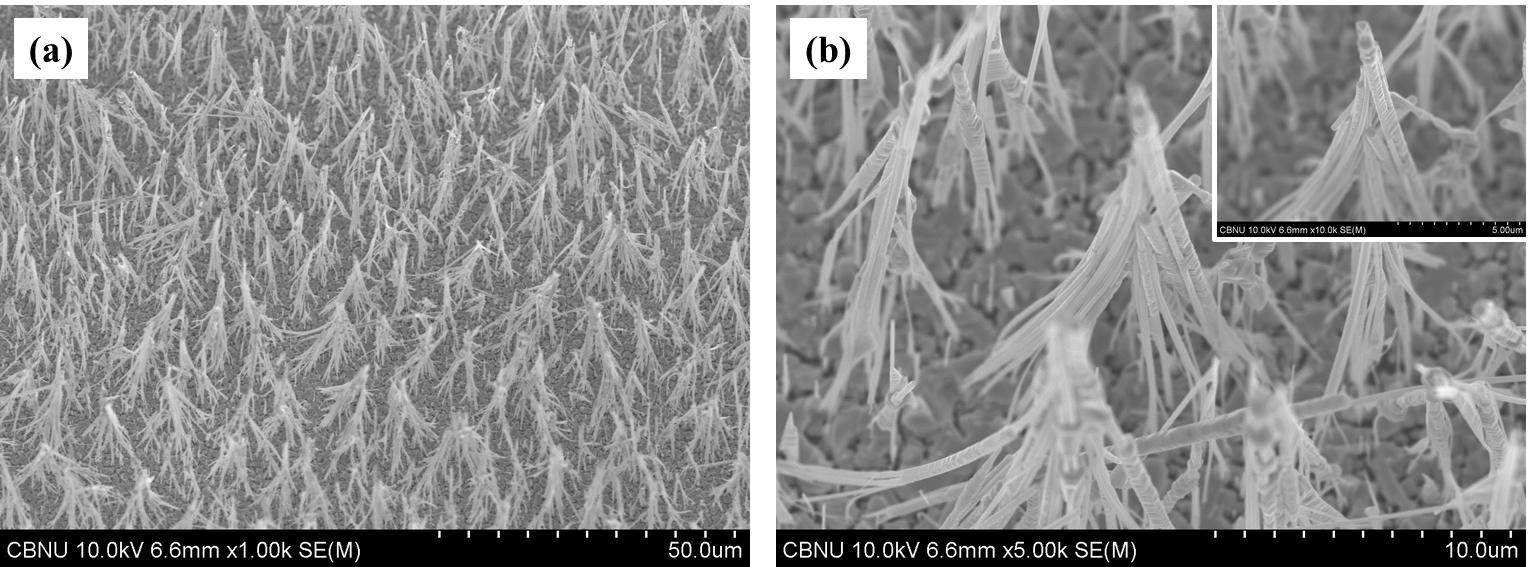


**Figure S2** | FE-SEM images of MOCVD-grown InAs NWs after the DTW process.

**S3. Uniformity of THz signals for InAs NWs**

In the initial stage of THz measurements on our InAs NWs, we confirmed the uniformity in the THz characteristics of InAs NWs over an entire 2-inch wafer. We randomly measured the THz signals at five different points on a 2-inch wafer. The shape and intensity of the THz signals were almost same. **Figure S3** shows (a) time-domain current signals and (b) THz spectra of the InAs NWs with the height of 10 μm, measured at five differnt points on a 2-inch wafer.

**
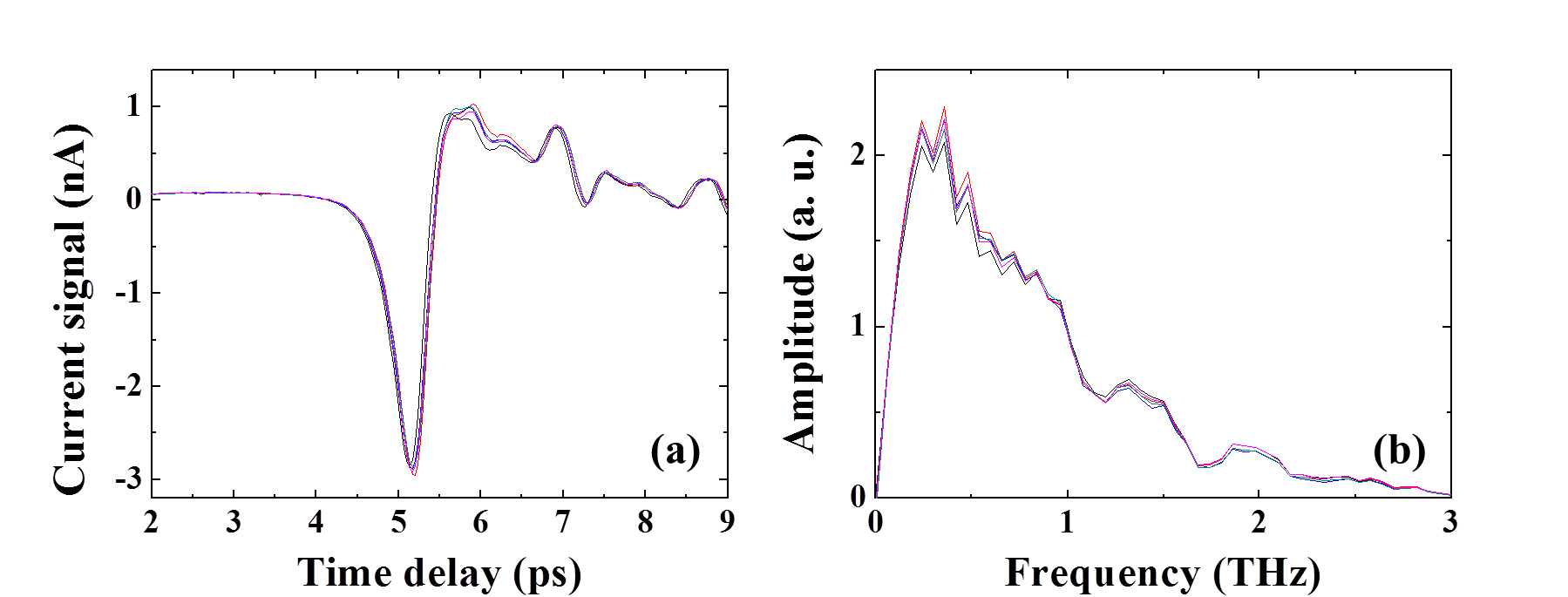
**

**Figure S3** | (a) Time-domain current signals and (b) THz spectra of the InAs NWs with the height of 10 μm, measured at five differnt points on a 2-inch wafer.
